# Protective effect of Astragalus membranaceus and Astragaloside IV in sepsis-induced acute kidney injury

**DOI:** 10.18632/aging.204189

**Published:** 2022-07-20

**Authors:** Jia-Long Tang, Meng Xin, Li-Chao Zhang

**Affiliations:** 1 Department of Pharmacy, Shanghai Municipal Hospital of Traditional Chinese Medicine, Shanghai University of Traditional Chinese Medicine, Shanghai, China

**Keywords:** sepsis-associated acute kidney injury, Astragalus membranaceus, Astragaloside IV, network pharmacology, PI3K

## Abstract

Background: Acute kidney injury (AKI) is the most common target organ damage in sepsis. Sepsis-associated AKI (SA-AKI) may be characterized by damage to the renal tubular epithelium. In this study, the pharmacological mechanisms of Astragalus membranaceus and its active monomer Astragaloside IV (AS-IV) were predicted based on a network pharmacology approach and validated both *in vitro* and *in vivo* using the SA-AKI model.

Method: We constructed an *in vivo* sepsis model using a mouse cecum ligation puncture (CLP) and HK-2 cells were treated with lipopolysaccharide (LPS) to mimic Gram (–) induced sepsis to assess the renal-protective efficacy of Astragalus membranaceus and AS-IV.

Results: The findings demonstrated that Astragalus membranaceus and AS-IV attenuate renal tubular injury in mice with polymicrobial sepsis, including vacuolization, loss of brush border, mitochondrial ultrastructural changes, and increased staining of kidney injury molecule-1 (KIM-1). AS-IV protected human proximal tubular epithelial (HK-2) cells against LPS induced cell viability loss. Both Astragalus membranaceus and AS-IV activated the PI3K/AKT pathway both *in vitro* and *in vivo*, as shown by Western blot and immunohistochemistry analysis.

Conclusion: The findings demonstrate that Astragalus membranaceus and AS-IV protect against sepsis-induced kidney tubular injury by activating the PI3K/AKT pathway.

## INTRODUCTION

Sepsis is defined as life-threatening organ dysfunction caused by a dysregulated immune response to infection, which means that sepsis is diagnosed when the body’s response to infection results in life-threatening damage to its tissues or organs [1]. Sepsis is now the leading cause of mortality among patients in intensive care units [2]. The most significant and common complication of sepsis is Acute kidney injury (AKI) [3]. Sepsis-associated AKI (SA-AKI) accounts for 45–70% of all AKI cases in foreign intensive care units, with a mortality rate of 60–80% in AKI patients requiring dialysis [4]. Therefore, preventing and controlling the occurrence of SA-AKI and reducing the morbidity and mortality of septic patients is another challenge is another important public health concern. It has been shown that in sepsis, inflammatory mediators originating from pathogens and activated immune cells can act on renal tubular epithelial cells via a Toll-like receptor 4 (TLR4)-dependent pathway, leading to mitochondrial dysfunction and tubular epithelial cell injury [5]. Therefore, it is presumed that the inflammatory response, tubular epithelial cell injury, and renal microcirculatory disorders are all involved in the development of SA-AKI. However, the pathogenesis of SA-AKI remains unknown.

Astragalus membranaceus (AM) has historically been utilized primarily as a Qi-tonifying in traditional Chinese medicine. The active ingredients contained in Astragalus membranaceus and related plants have been investigated extensively [6]. Astragalus membranaceus is distributed across China, with the medicinal Astragalus most widely used Shanxi, Heilongjiang, and Inner Mongolia. Astragalus is rich in chemical constituents and much research has been conducted to isolate and purify the saponin, polysaccharide, and flavonoid components of Astragalus as well as their pharmacological effects [7, 8]. Astragalus has more than fifty saponins that exist in a discrete state. Astragaloside I/IV, Mauroisoflavone glycosides and Astragaloside Isoflavan are all found in abundance in Astragaloside membranaceus [9]. Astragaloside membranaceus possesses favorable pharmacological properties and plays a unique role in anti-inflammation, cardiovascular improvement, and neuroprotection [9]. Additionally, Astragalus contains trace elements such as proteins, amino acids, monosaccharides, and folic acid in trace levels. However, research on these trace elements is scarce [10]. Although Astragalus is of the most extensively used herbal remedies for the treatment of kidney diseases, the material basis for its action and mechanism of action are unknown. Certain herbal remedies have demonstrated significant benefits and high efficacy in inhibiting the inflammatory response, programmed cell death, i.e., apoptosis and oxidative stress. The therapeutic effects of herbal medicine have been extensively validated in animal models and even in humans [11]. However, the effects and specific mechanisms of Astragalus in the treatment of SA-AKI have not been reported.

The theory of network pharmacology was proposed by Hopkins in 2007 based on the scope of systems biology and pharmacology. The concept is based on multi-target research, which involves collecting and organizing data on target drugs, constructing bio-visualization networks, and analyzing the interactions between drugs and genes, targets, and diseases to reveal the mechanism of action from a systematic and holistic manner [12]. The mechanism of action of Chinese medicines in regulating various signaling pathways has now been applied to the study of the material basis for drug efficacy and mechanism of action of Chinese medicines. In recent years, as multi-omics integration research has advanced, the acquisition of targets in network pharmacology has largely relied on directly related experimental data, significantly improving the accuracy of network pharmacology mechanisms and pharmacodynamic substance bases. Additionally, as big data analysis and molecular docking technology advance, the accuracy of the network pharmacology will improve further [13]. In this study, we investigated the mechanism of Astragalus membranaceus’s anti-SA-AKI effect using network pharmacology. This was achieved by first investigating the active components, targets of action, and mechanisms of Astragalus membranaceus against SA-AKI.

## MATERIALS AND METHODS

### Chemicals and reagents

LPS (*E. coli* 0111: B4) was obtained from Millipore Sigma. Astragalus membranaceus was purchased from Kang Qiao Traditional Chinese Medicine Decoction Pieces Co. Ltd. Astragaloside IV (purity above 98%) was purchased from Xi’an Sobeo Pharmaceutical Technology Co., Ltd. The antibodies used for Western blotting, PI3K (#4255) and Phospho-PI3K p85 (Tyr458) (#17366) were purchased from Cell Signaling Technology. AKT (A17909) and Phospho-AKT (Ser473) (AP0637) were purchased from ABclonal.

### Water extract of Astragalus membranaceus

Astragalus slices (500 g) were weighed, distilled water was added in a ration of 1:10, soaked for 2–3 h, then decocted and extracted 3 times at 90°C for 1.5 h. The decoction was filtered twice with gauze and the filtrate combined. The filtrate was concentrated to a viscous liquid using a rotary evaporator at 50°C, poured into trays, and dried in a water bath set at 65°C to form an infusion. The infusion was frozen for two days at −80°C and then lyophilized to form a powder in a freeze dryer.

### Animals

Male C57BL/6J mice (18–22 g) were purchased from the Changzhou Cavens Laboratory Animal Co., China. All mice were housed in an automated 12-hour dark-light cycle at a controlled temperature of 22°C ± 2°C and relative humidity of 50–60%, with free access to standard dry feed and tap water. All animal experiments were conducted in accordance with the NIH Guide for the Care and Use of Laboratory Animals (National Academies Press, 2011) and were approved by the Naval Medical University Committee on Animal Care (EC11-055).

### Sepsis model

Sepsis was induced by CLP as previously described [14]. Isoflurane was used to completely anesthetize the mice, and a midline abdominal incision was made. The ileocecal valve was ligated on the distal 3/4 of the cecum. in a sterile environment, the cecum was perforated twice with a 21-gauge needle, and a droplet of feces was extruded from the perforations to induce polymicrobial peritonitis. Sutures were placed in two layers along the abdomen wall and 1 ml of a 0.9% sodium chloride solution was administered for fluid resuscitation. The sham group underwent laparotomy and bowel manipulation without ligation or perforation. Following recovery from anesthesia, all mice had unrestricted access to food and water. There were three groups of six animals each for Astragalus membranaceus: the sham-operated group (Sham group), the sepsis model group (CLP group), and treatment groups with Astragalus membranaceus (250 mg/kg). Astragalus membranaceus was administrated orally every day before CLP. There were four groups of six animals each in the AS-IV study, a sham group, a CLP group, and two treatment groups: AS-IV low dose (5 mg/kg) and AS-IV high dose (10 mg/kg). AS-IV was administrated orally every day before CLP.

### Kidney injury study

Kidney tissues were cut into 5 μm sections and stained with H&E, PAS, and nitrotyrosine reagents, as well as KIM-1 antibody and Phospho-AKT antibody. The kidney injury score is as follows: 0, normal; 1, <10%; 2, 10–25%; 3, 25–50%; 4, 50–75%; 5, 75–100% of affected kidney areas obtained from mice exposed to sham or CLP surgery. Tubular atrophy or dilatation, loss of the brush border, vacuolization, epithelial cell shedding, and denuded tubular basement membrane were some of the criteria.

### Cell culture

HK-2 cells (CRL-2190, ATCC, Rockefeller, MD, USA) were maintained in RPMI1640 (31800022, Gibco, NY, USA) supplemented with 10% v/v FBS, 100 U/ml penicillin, and 100 μg/ml streptomycin (Thermo Fisher Scientific, Waltham, MA, USA) in humidified incubators at 37°C and 5% CO_2_.

### Bioinformatics analysis

The TCMSP server consisted of a system-level pharmacology database with a flexible, user-friendly web interface. To gain a better understanding of the complex, network targets genes and diseases were constructed and analyzed using Cytoscape 3.0.

### Western blotting

The protein samples were electrophoresed in 8–12% SDS-PAGE gels and transferred onto nitrocellulose membranes (Amersham). The membranes were blocked with 5% BSA in PBS for 1 hour at room temperature and then probed overnight at 4°C with primary antibodies (1:1000 dilution). The membranes were then incubated with secondary antibodies (1:10,000 dilution) for 50 min at room temperature. Finally, Odyssey (LI-COR, Lincoln, NE, USA) and the relative protein expression were calculated from gray-scale blots using ImageJ software.

### Statistics

GraphPad Prism 9.0.1 was used to perform statistical analysis and data were expressed as mean ± SEM. Statistical significance was determined using Student’s *t*-test or ANOVA. *P* < 0.05 was considered statistically significant.

### Data statement

Our data are available. Please contact the corresponding author for requirement. More detailed Materials and Methods are in the Supplementary Materials and Methods.

## RESULTS

**Figure 1:**
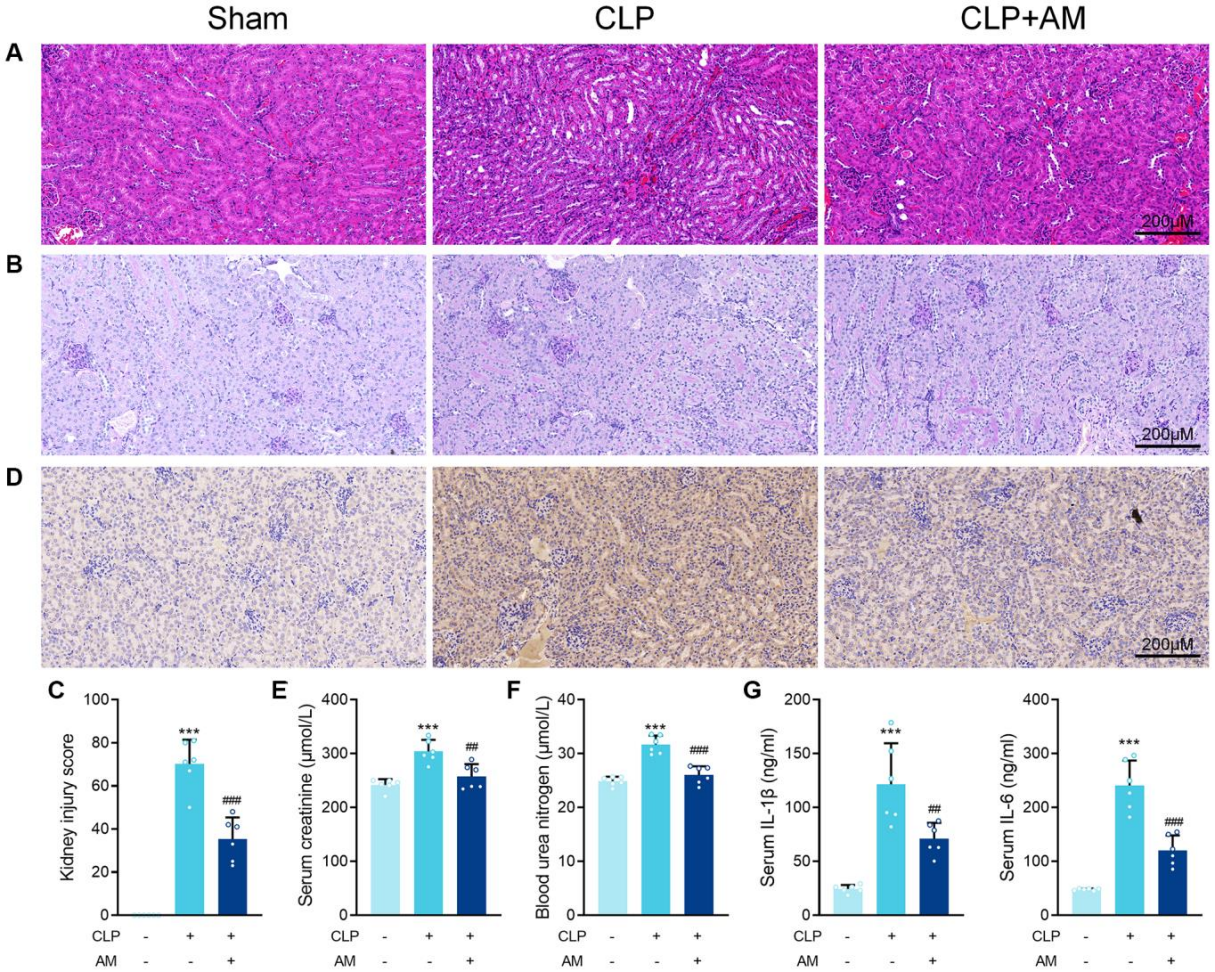
**Astragalus membranaceus protects against renal injury in CLP-induced sepsis.** (**A**) Representative images of H&E staining of kidney sections obtained at 24 hours after CLP surgery following treatment with (250 mg/kg) Astragalus membranaceus. (**B**) Representative images of periodic acid Schiff’s staining reagent depicting basement membrane and brush border in kidney sections. (**C**) Semiquantitative analysis of tubular injury. (**D**) Representative images showing KIM-1 immunohistochemical staining results in kidney tissues. Serum creatinine (**E**) and Blood urea nitrogen (**F**) in mice were measured. (**G**) Serum IL-6 and IL-1β concentrations were measured by ELISA. Data are presented as the mean ± SEM (*n* = 6 per group, ^***^P < 0.001 compared to Sham group, ^##^*P* < 0.01, ^###^*P* < 0.001 compared to CLP group).

Astragalus membranaceus protects against renal injury in CLP-induced sepsis. In this study, the CLP model, a widely accepted mouse model of sepsis, was used to induce SA-AKI in C57BL/6J mice. Histopathological examination revealed that 24 hours after CLP surgery, the kidney tissue interstitial edema in the CLP mice was obvious, with diffuse neutrophil infiltration and severe glomerular wrinkling, while the tubular lumen was occluded, some cells were swollen and vacuolated, and the tubular cystic lumen was dilated. Further treatment with Astragalus membranaceus (AM) improved focal tubular damage, tubular swelling, and vacuolar degeneration as well as inflammatory cell infiltration and interstitial edema to varying degrees in mice kidney tissues (Figure 1A and 1B). Consistently, septic mice after AM treatment exhibited a markedly reduced kidney injury score (Figure 1C). Immunohistochemical analysis of kidney sections from treated septic mice revealed decreased staining for kidney injury molecule-1 (KIM-1) at the ends of renal tubular epithelial cells (Figure 1D). Furthermore, blood urea nitrogen (BUN) and creatinine levels, indicative of kidney dysfunction, were markedly decreased in mice of the treatment groups, suggesting preserved renal function for AM (Figure 1E and 1F). ELISA results confirmed that IL-6 and IL-1β concentrations in serum were deceased after AM treatment (Figure 1G).

### The effect of Astragalus membranaceus active ingredients on LPS-induced damage on HK-2 cell line

**Figure 2:**
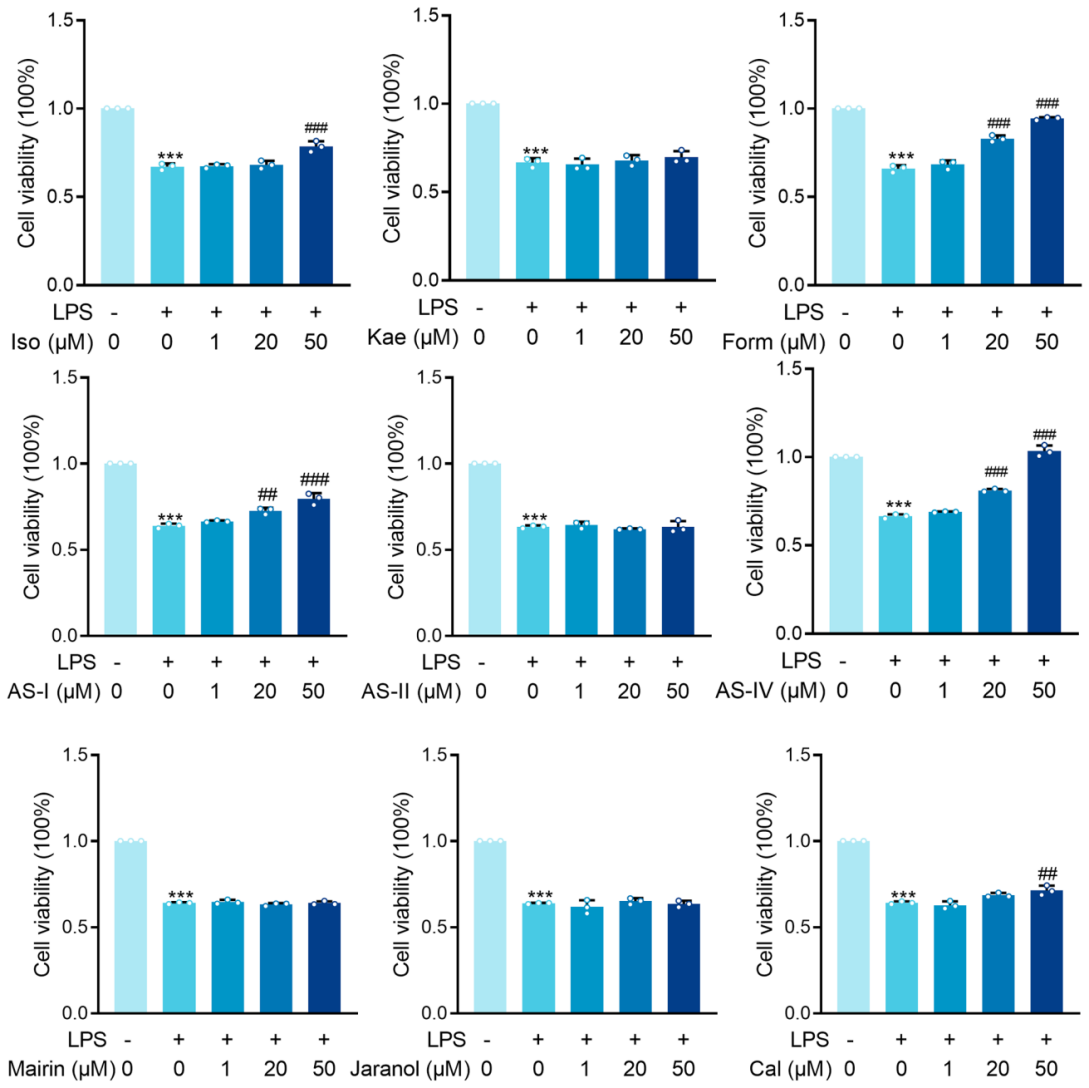
**MTT results for isorhamnetin (Iso), kaempferol (Kae), formononetin (Form), Astragaloside I (AS-I), Astragaloside II (AS-II), Astragaloside IV (AS-IV), Mairin, Jaranol and Calycosin (Cal) treatment on renal epithelial cells HK-2 cells incubated with LPS (1 μg/ml) for 6 hours.** Data are presented as the mean ± SEM (*n* = 3 per group, ^***^P < 0.001 compared to Control group, ^##^*P* < 0.01, ^###^*P* < 0.001 compared to LPS group).

Given that sepsis’s primary target is proximal tubular epithelial cells, HK-2 cells were treated with LPS to mimic Gram (–) induced sepsis-associated tubular injury. A total of 144 Astragalus-related compounds were identified through a database search. The TCMSP database was examined for OB and DL values of each compound, and 42 compounds (28.6%) had OB values greater than 30%, 50 compounds (40%) had DL values greater than 0.18, and 22 compounds (31.4%) had optimum OB and DL values. Astragalus saponins were excluded from the study due to their failure to meet the OB and DL thresholds. However, because these components are the most often reported active ingredients in the pharmacological action of Astragalus, they were included in this study. To screen for anti-SA-AKI activity, the final 26 monomers from Astragalus were collected (Table 1). We discovered nine compounds, including isorhamnetin, kaempferol, mangosteen, and astragaloside, that have been reported in “sepsis” or “acute kidney injury” and have been verified by the official website of the China Academy of Food and Drug Control. The nine compounds were screened further using LPS-induced HK-2 cells. The MTT assay was used to examine the effects of isorhamnetin (Iso), kaempferol (Kae), formononetin (Form), astragaloside I (AS-I), astragaloside II (AS-II), astragaloside IV (AS-IV), Mairin, Jaranol, and Calycosin (Cal) on LPS-induced HK-2 cell injury *in vitro* (Figure 2).

**Table 1:** Active ingredients of Astragalus membranaceus.

**No.**	**Compound**	**MOL ID**	**OB**	**DL**
C1	Mairin	MOL000211	55.38	0.78
C2	kaempferol	MOL000422	41.88	0.24
C3	Jaranol	MOL000239	50.83	0.29
C4	isorhamnetin	MOL000354	49.60	0.31
C5	isomucronulatol-7,2′-di-O-glucosiole	MOL000439	49.28	0.62
C6	isoflavanone	MOL000398	109.99	0.30
C7	hederagenin	MOL000296	36.91	0.75
C8	formononetin	MOL000392	69.67	0.21
C9	astragaloside IV	MOL000407	22.50	0.15
C10	astragaloside I	MOL000401	46.79	0.11
C11	astragaloside II	MOL000403	46.06	0.13
C12	astragaloside III	MOL000405	31.83	0.10
C13	Quercetin	MOL000098	46.43	0.28
C14	FA	MOL000433	68.96	0.71
C15	Calycosin	MOL000417	47.75	0.24
C16	Bifendate	MOL000387	31.10	0.67
C17	9,10-dimethoxypterocarpan-3-O-β-D-glucoside	MOL000379	36.74	0.92
C18	https://tcmspw.com/molecule.php?qn=378	MOL000378	74.69	0.30
C19	5′-hydroxyiso-muronulatol-2′,5′-di-O-glucoside	MOL000374	41.72	0.69
C20	3,9-di-O-methylnissolin	MOL000371	53.74	0.48
C21	(6aR,11aR)-9,10-dimethoxy-6a,11a-dihydro-6H-benzofurano [3,2-c] chromen-3-ol	MOL000380	64.26	0.42
C22	(3R)-3-(2-hydroxy-3,4-dimethoxyphenyl) chroman-7-ol	MOL000438	67.67	0.26
C23	1,7-Dihydroxy-3,9-dimethoxy pterocarpene	MOL000442	39.05	0.48
C24	(3S,8S,9S,10R,13R,14S,17R)-10,13-dimethyl-17-[(2R,5S)-5-propan-2-yloctan-2-yl]-2,3,4,7,8,9,11,12,14,15,16,17-dodecahydro-1H-cyclopenta[a]phenanthren-3-ol	MOL000033	36.23	0.78
C25	β-sitosterol	MOL001987	33.94	0.70
C26	(+)-Medicarpin	MOL011076	60.46	0.34

### The effect of LPS on HK-2 cells

LPS significantly inhibited the survival rate of HK-2 cells (*P* < 0.001). Astragaloside I (AS-I) and Astragaloside IV (AS-IV) demonstrated dose-dependent protective effects at 20 μM versus 50 μM concentrations (AS-I: *P* < 0.01, *P* < 0.001; Form: *P* < 0.001, *P* < 0.001; AS-IV: *P* < 0.001, *P* < 0.001). At 50 μM Iso and Cal showed significant protection (*P* < 0.001). In contrast, treatment of HK-2 cells with Kae, AS-II, Mairin, and Jaranol did not result in any significant changes in the LPS-induced cytotoxicity (Figure 2). Our results demonstrated that AS-I, AS-IV, Iso, and Cal all exerted protective effects against LPS-induced HK-2 cytotoxicity in renal epithelial cells at 50 μM concentration, with AS-IV exhibiting the most pronounced protective effect. Therefore, we further investigated the mechanism through which AS-IV protects against septic acute kidney injury.

### AS-IV protects against renal injury in CLP-induced sepsis

**Figure 3:**
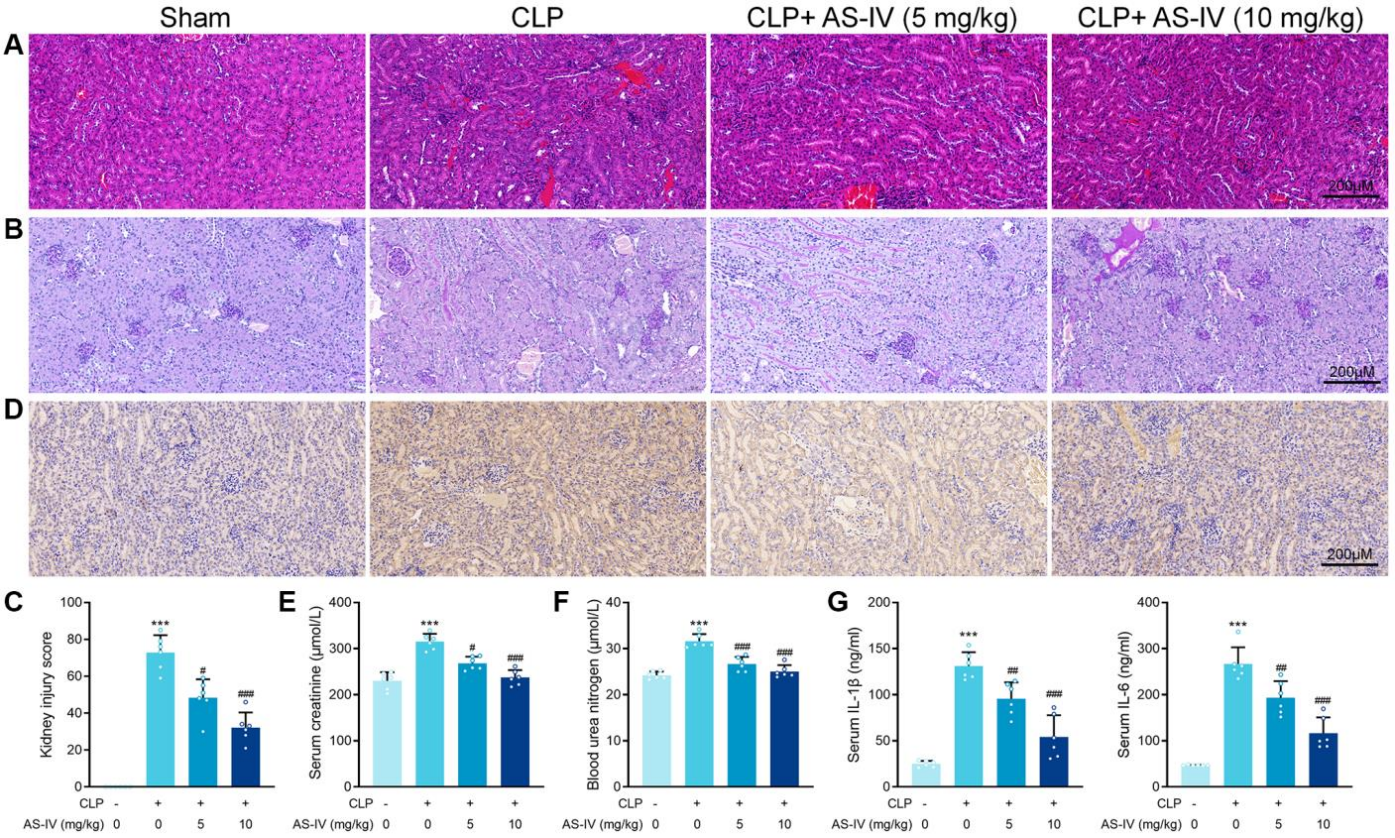
**AS-IV protects against renal injury in CLP-induced sepsis.** (**A**) Representative images of H&E staining of tubular epithelial cells of kidney sections obtained at 24 hours after CLP surgery following treatment with low (5 mg/kg) or high dosage (10 mg/kg) AS-IV. (**B**) Representative images of periodic acid Schiff’s staining reagent depicting basement membrane and brush border in the kidney sections. (**C**) Semiquantitative analysis of tubular injury. (**D**) Representative images showing KIM-1 immunohistochemical staining of the apical side of tubular epithelial cells. Serum creatinine (**E**) and Blood urea nitrogen (**F**) in mice were measured. (**G**) Serum IL-6 and IL-1β concentrations were measured by ELISA. Data are presented as the mean ± SEM (*n* = 6 per group, ^***^P < 0.001 compared to Sham group, ^#^*P* < 0.05, ^##^*P* < 0.01, ^###^*P* < 0.001 compared to CLP group).

The China Pharmacopeia 2015 specifies that the content of AS-IV in Astragalus should not be less than 0.040%. Using HPLC analysis, we determined the AS-IV content of Astragalus to be 0.147%, which met the quality standard requirements stipulated in the China Pharmacopeia 2015 (Supplementary Figure 1). To determine the protective effect of AS-IV on SA-AKI-induced renal damage, we subjected mice to CLP or sham surgery. The treatment group was divided into two groups, low-dose (i.p. 5 mg/kg) and high-dose (i.p. 10 mg/kg). Similarly, the AS-IV 5 mg/kg and 10 gm/kg groups had considerably fewer intracellular vacuoles (Figure 3A).

Additionally, the treatment groups showed significant loss of brush border in the renal proximal tubular epithelial cells caused by CLP (Figure 3B). After AS-IV treatment, mice showed a markedly decrease kidney injury score (Figure 3C). Immunohistochemical analysis revealed decreased staining of KIM-1 on the apical side of renal tubular epithelial cells in kidney sections from treated septic mice (Figure 3D). Next, BUN and creatinine levels were markedly decreased in mice of the treatment groups, indicating preserved renal function for AS-IV (Figure 3E and 3F). ELISA results confirmed that IL-6 and IL-1β concentrations in serum were deceased after AS-IV treatment (Figure 3G).

### Astragalus membranaceus and AS-IV for SA-AKI target prediction

**Figure 4:**
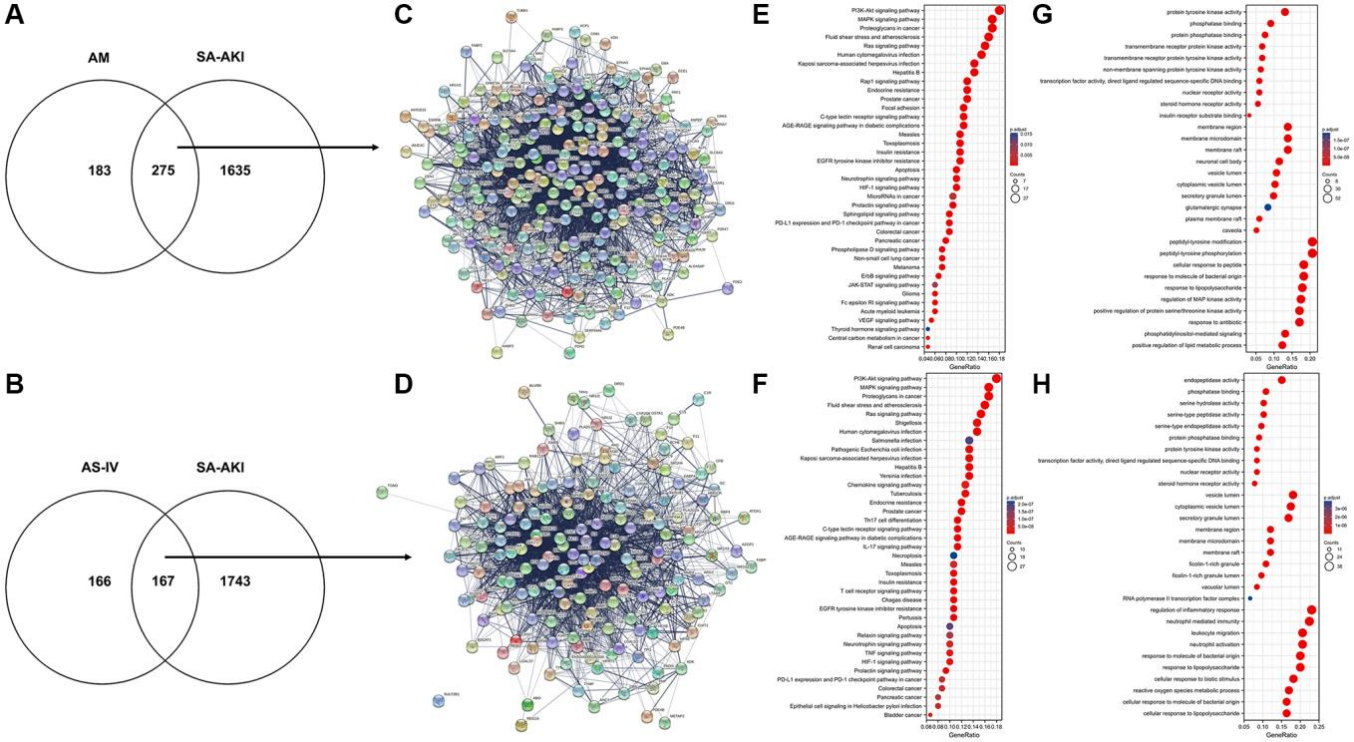
**Astragalus membranaceus and AS-IV for SA-AKI target prediction.** (**A**) Venn diagram of Astragalus membranaceus active compounds-related targets and SA-AKI-related targets. (**B**) Venn diagram of AS-IV active compounds-related targets and SA-AKI-related targets. (**C**) PPI network of potential targets of Astragalus membranaceus anti-SA-AKI. (**D**) PPI network of potential targets of AS-IV anti-SA-AKI. (**E**) KEGG pathway analysis of potential targets of Astragalus membranaceus anti-SA-AKI. (**F**) KEGG pathway analysis of potential targets of AS-IV anti-SA-AKI. (**G**) GO analysis of potential targets of Astragalus membranaceus anti-SA-AKI. (**H**) GO analysis potential targets of AS-IV anti-SA-AKI.

The therapeutic target database (TTD), Genecards, the Comparative Toxicogenomics Database (CTD), and the CTD were searched using the terms “sepsis” and “acute kidney damage (AKI). Genecards and the Comparative Toxicogenomics Database (CTD) were used to search for relevant disease targets, and the CTD database was used to identify experimentally validated disease targets designated as “marker/mechanism” or “therapeutic”. The CTD database selects targets with a high degree of confidence that has been experimentally validated with “marker/mechanism” or “therapeutic” as targets for research. Finally, 2498 sepsis-related genes and 6746 AKI-related genes were retrieved from the GeneCards database, while 48 sepsis-related genes and 120 AKI-related genes were retrieved from the CTD database. A total of 1938 targets were obtained after a comprehensive screening of all targets to eliminate duplicates, and the DAVID database validated a total of 1910 targets related to sepsis and acute kidney injury in humans. All genes were imported into the DAVID database, and the relevant genes were exported by selecting and clicking “show gene lis”. GO enrichment and KEGG pathway enrichment analyses were performed on the disease targets via the DAVID database, using *P* < 0.01 and False discovery rate (False discovery rate (FDR) <0.01 as the screening thresholds. A total of 686 entries for biological process (BP), 104 for cellular component (CC), and 121 for molecular function (MF) were obtained. The top 20 entries are displayed in Tables 2 and 3 below, are ordered by the magnitude of their P and FDR values. SA-AKI was predicted to be involved in biological processes such as the inflammatory response, immune response, intrinsic immune response, response to LPS, response to hypoxic factors, etc. The cellular components were involved in extracellular gaps, extracellular regions, molecular functions including protein binding, receptor binding, enzyme binding, etc. Astragalus and SA-AKI targets were intersected by Venn to obtain a total of 275 core targets (Figure 4A). AS-IV and SA-AKI targets were intersected by Venn to obtain a total of 167 core targets (Figure 4B). We further examined the relationship between the bio-active ingredients and the core targets, to construct an interaction network between the two (Supplementary Figure 2). A total of 26 active ingredients of Astragalus were identified to be critical to the core target. The high-degree nodes in the network exhibited a greater number of component-target-pathway interactions, which were likely to play a critical role in renal protective AKI. Following that, the Astragalus Target-SA-AKI Target-PPI Network input the compound targets into TCMSP and String databases. There were 275 nodes and 741 edges in the Astragalus Target-SA-AKI Target-PPI Network (Figure 4C). Similarly, the AS-IV Target-SA-AKI Target-PPI Network Input the compound targets into TCMSP and String databases. There were 167 nodes and 2413 edges in the AS-IV Target-SA-AKI Target-PPI Network (Figure 4D). We imported the 254 target genes of Astragalus anti-SA-AKI and the 167 target gene sets of AS-IV anti-SA-AKI into the DAVID version 6.7 database for Gene ontology analysis and KEGG pathway enrichment analysis, using three panels typically included in GO analysis. For Astragalus, a total of 241 MF entries, 3045 BF entries, 123 CC entries, and 169 KEGG entries were obtained. Following that, we screened at *P* < 0.01 and FDR <0.01 to extract significant card values and discovered 2028 entries under BP, 63 entries under CC, and 132 entries under MF. Figure 4E and Table 4 show the top 10 entries selected for visualization. As shown in Figure 4F and Table 5, 140 KEGG entries were selected for visualization. For AS-IV, 168 entries were obtained under MF, 2661 entries under BP, 64 entries under CC, and 147 entries under KEGG. We used *P* < 0.01 and FDR <0.01 as significant card values and obtained 1667 entries under BP, 43 entries under CC, and 89 entries under MF. The top 10 entries were selected for visualization, as shown in Figure 4G and Table 6. 114 entries under KEGG were selected for visualization, as shown in Figure 4H and Table 7.

**Table 2:** GO enrichment analysis of disease mechanisms in SA-AKI.

**Ontology**	**ID**	**Description**	***P* value**	**FDR**
BP	0006954	inflammatory response	4.41E-98	2.71E-94
BP	0042493	response to drug	9.21E-58	2.83E-54
BP	0006955	immune response	2.16E-55	4.43E-52
BP	0045087	innate immune response	1.08E-48	1.66E-45
BP	0032496	response to lipopolysaccharide	2.50E-46	3.08E-43
BP	0001666	response to hypoxia	5.70E-43	5.19E-40
BP	0043066	negative regulation of apoptotic process	5.90E-43	5.19E-40
BP	0008284	positive regulation of cell proliferation	7.95E-41	6.11E-38
BP	0051092	positive regulation of NF-kappaB transcription factor activity	3.14E-39	2.15E-36
BP	0050900	leukocyte migration	1.88E-37	1.16E-34
BP	0071222	cellular response to lipopolysaccharide	4.05E-36	2.26E-33
BP	0045944	positive regulation of transcription from RNA polymerase II promoter	1.28E-35	6.58E-33
BP	0007165	signal transduction	1.86E-34	8.82E-32
BP	0070374	positive regulation of ERK1 and ERK2 cascade	6.05E-33	2.66E-30
BP	0007568	aging	1.46E-32	6.01E-30
BP	0045471	response to ethanol	2.73E-32	1.05E-29
BP	0010628	positive regulation of gene expression	2.58E-30	9.35E-28
BP	0045766	positive regulation of angiogenesis	2.86E-30	9.79E-28
BP	0006915	apoptotic process	1.04E-29	3.36E-27
BP	0050731	positive regulation of peptidyl-tyrosine phosphorylation	3.21E-29	9.89E-27
CC	0005615	extracellular space	3.41E-141	2.46E-138
CC	0005576	extracellular region	1.01E-97	3.64E-95
CC	0070062	extracellular exosome	5.69E-82	1.37E-79
CC	0009986	cell surface	1.58E-54	2.85E-52
CC	0005886	plasma membrane	3.42E-54	4.94E-52
CC	0009897	external side of plasma membrane	1.55E-51	1.87E-49
CC	0005829	cytosol	1.28E-44	1.32E-42
CC	0045121	membrane raft	2.26E-37	2.04E-35
CC	0005925	focal adhesion	4.75E-33	3.82E-31
CC	0005887	integral component of plasma membrane	7.79E-32	5.63E-30
CC	0016020	membrane	1.32E-28	8.68E-27
CC	0072562	blood microparticle	4.56E-26	2.75E-24
CC	0031012	extracellular matrix	4.24E-24	2.36E-22
CC	0031093	platelet alpha granule lumen	8.12E-21	4.20E-19
CC	0043202	lysosomal lumen	2.13E-18	1.03E-16
CC	0043234	protein complex	1.55E-17	6.82E-16
CC	0048471	perinuclear region of cytoplasm	1.60E-17	6.82E-16
CC	0005764	lysosome	2.36E-17	9.47E-16
CC	0005796	Golgi lumen	1.88E-14	7.16E-13
CC	0005911	cell-cell junction	5.39E-13	1.95E-11
MF	0005515	protein binding	7.49E-56	1.32E-52
MF	0005102	receptor binding	1.57E-42	1.38E-39
MF	0019899	enzyme binding	1.75E-31	1.03E-28
MF	0042803	protein homodimerization activity	2.56E-31	1.13E-28
MF	0042802	identical protein binding	7.35E-29	2.59E-26
MF	0005125	cytokine activity	3.02E-27	8.89E-25
MF	0008083	growth factor activity	1.62E-20	4.09E-18
MF	0002020	protease binding	5.13E-20	1.13E-17
MF	0004872	receptor activity	7.69E-19	1.51E-16
MF	0001618	virus receptor activity	2.08E-18	3.67E-16
MF	0019901	protein kinase binding	3.64E-18	5.83E-16
MF	0005088	Ras guanyl-nucleotide exchange factor activity	1.84E-16	2.70E-14
MF	0005179	hormone activity	2.08E-16	2.83E-14
MF	0008201	heparin binding	6.82E-16	8.60E-14
MF	0001948	glycoprotein binding	7.96E-16	9.36E-14
MF	0004252	serine-type endopeptidase activity	3.97E-15	4.38E-13
MF	0046934	phosphatidylinositol-4,5-bisphosphate 3-kinase activity	1.00E-14	1.04E-12
MF	0004713	protein tyrosine kinase activity	2.83E-14	2.77E-12
MF	0005164	tumor necrosis factor receptor binding	1.30E-13	1.20E-11
MF	0005178	integrin binding	1.36E-13	1.20E-11

**Table 3:** KEGG enrichment analysis of disease mechanisms in SA-AKI.

**Ontology**	**ID**	**Description**	***P* value**	**FDR**
KEGG_PATHWAY	hsa04668	TNF signaling pathway	5.13E-34	8.61E-32
KEGG_PATHWAY	hsa05145	Toxoplasmosis	1.38E-31	1.16E-29
KEGG_PATHWAY	hsa04064	NF-kappa B signaling pathway	6.61E-31	3.39E-29
KEGG_PATHWAY	hsa04060	Cytokine-cytokine receptor interaction	6.61E-31	3.39E-29
KEGG_PATHWAY	hsa04610	Complement and coagulation cascades	1.01E-30	3.39E-29
KEGG_PATHWAY	hsa05200	Pathways in cancer	2.62E-30	7.33E-29
KEGG_PATHWAY	hsa04380	Osteoclast differentiation	6.57E-30	1.58E-28
KEGG_PATHWAY	hsa05152	Tuberculosis	1.99E-29	4.17E-28
KEGG_PATHWAY	hsa04620	Toll-like receptor signaling pathway	2.30E-27	4.29E-26
KEGG_PATHWAY	hsa05140	Leishmaniasis	5.90E-27	9.91E-26
KEGG_PATHWAY	hsa04640	Hematopoietic cell lineage	1.54E-26	2.35E-25
KEGG_PATHWAY	hsa05164	Influenza A	4.85E-25	6.79E-24
KEGG_PATHWAY	hsa04062	Chemokine signaling pathway	6.48E-25	8.37E-24
KEGG_PATHWAY	hsa04660	T cell receptor signaling pathway	3.49E-23	4.19E-22
KEGG_PATHWAY	hsa05133	Pertussis	1.03E-22	1.15E-21
KEGG_PATHWAY	hsa05142	Chagas disease (American trypanosomiasis)	1.17E-22	1.22E-21
KEGG_PATHWAY	hsa05161	Hepatitis B	2.31E-22	2.28E-21
KEGG_PATHWAY	hsa05162	Measles	7.21E-22	6.73E-21
KEGG_PATHWAY	hsa05321	Inflammatory bowel disease (IBD)	1.36E-21	1.21E-20

**Table 4:** GO enrichment analysis of potentially relevant targets of Astragalus anti-SA-AKI.

**Ontology**	**ID**	**Description**	**GeneRatio**	**BgRatio**	***p* value**	**FDR**	***q* value**
BP	GO:0018108	peptidyl-tyrosine phosphorylation	52/252	363/18670	2.78E-38	1.01E-34	4.03E-35
BP	GO:0018212	peptidyl-tyrosine modification	52/252	366/18670	4.27E-38	1.01E-34	4.03E-35
BP	GO:0002237	response to molecule of bacterial origin	46/252	343/18670	1.62E-32	2.56E-29	1.02E-29
BP	GO:0032496	response to lipopolysaccharide	45/252	330/18670	3.94E-32	4.66E-29	1.86E-29
BP	GO:0043405	regulation of MAP kinase activity	44/252	337/18670	1.35E-30	1.27E-27	5.09E-28
BP	GO:1901653	cellular response to peptide	46/252	385/18670	2.97E-30	2.34E-27	9.35E-28
BP	GO:0046677	response to antibiotic	43/252	327/18670	4.90E-30	3.32E-27	1.32E-27
BP	GO:0071902	positive regulation of protein serine/threonine kinase activity	43/252	334/18670	1.20E-29	7.11E-27	2.84E-27
BP	GO:0045834	positive regulation of lipid metabolic process	31/252	146/18670	2.11E-28	1.11E-25	4.42E-26
BP	GO:0048015	phosphatidylinositol-mediated signaling	33/252	181/18670	6.82E-28	3.23E-25	1.29E-25
CC	GO:0045121	membrane raft	35/253	315/19717	9.60E-23	1.99E-20	1.34E-20
CC	GO:0098857	membrane microdomain	35/253	316/19717	1.07E-22	1.99E-20	1.34E-20
CC	GO:0098589	membrane region	35/253	328/19717	3.72E-22	4.61E-20	3.10E-20
CC	GO:0031983	vesicle lumen	27/253	339/19717	4.05E-14	3.76E-12	2.53E-12
CC	GO:0060205	cytoplasmic vesicle lumen	26/253	338/19717	2.77E-13	2.06E-11	1.39E-11
CC	GO:0034774	secretory granule lumen	25/253	321/19717	6.18E-13	3.83E-11	2.58E-11
CC	GO:0044853	plasma membrane raft	15/253	109/19717	9.65E-12	5.13E-10	3.45E-10
CC	GO:0043025	neuronal cell body	29/253	497/19717	1.11E-11	5.15E-10	3.47E-10
CC	GO:0005901	caveola	13/253	80/19717	2.76E-11	1.14E-09	7.68E-10
CC	GO:0098978	glutamatergic synapse	21/253	349/19717	5.20E-09	1.93E-07	1.30E-07
MF	GO:0004713	protein tyrosine kinase activity	33/253	134/17697	1.09E-31	7.11E-29	4.46E-29
MF	GO:0004715	non-membrane spanning protein tyrosine kinase activity	16/253	46/17697	1.28E-18	4.16E-16	2.61E-16
MF	GO:0004714	transmembrane receptor protein tyrosine kinase activity	17/253	62/17697	1.06E-17	2.29E-15	1.44E-15
MF	GO:0004879	nuclear receptor activity	15/253	47/17697	7.02E-17	9.13E-15	5.72E-15
MF	GO:0098531	transcription factor activity, direct ligand regulated sequence-specific DNA binding	15/253	47/17697	7.02E-17	9.13E-15	5.72E-15
MF	GO:0019199	transmembrane receptor protein kinase activity	17/253	79/17697	9.21E-16	9.98E-14	6.25E-14
MF	GO:0019902	phosphatase binding	23/253	185/17697	2.27E-15	2.11E-13	1.32E-13
MF	GO:0003707	steroid hormone receptor activity	14/253	56/17697	3.54E-14	2.87E-12	1.80E-12
MF	GO:0019903	protein phosphatase binding	19/253	140/17697	1.33E-13	9.57E-12	6.00E-12
MF	GO:0043560	insulin receptor substrate binding	8/253	11/17697	2.48E-13	1.61E-11	1.01E-11

**Table 5:** KEGG enrichment analysis of potentially relevant targets of Astragalus anti-SA-AKI.

**Ontology**	**ID**	**Description**	**GeneRatio**	**BgRatio**	***p* value**	**FDR**	***q* value**
KEGG	hsa05418	Fluid shear stress and atherosclerosis	24/150	139/8076	3.87E-17	9.95E-15	4.57E-15
KEGG	hsa05205	Proteoglycans in cancer	25/150	205/8076	3.74E-14	4.81E-12	2.21E-12
KEGG	hsa05215	Prostate cancer	18/150	97/8076	1.19E-13	9.19E-12	4.22E-12
KEGG	hsa01522	Endocrine resistance	18/150	98/8076	1.43E-13	9.19E-12	4.22E-12
KEGG	hsa01521	EGFR tyrosine kinase inhibitor resistance	16/150	79/8076	7.08E-13	2.60E-11	1.19E-11
KEGG	hsa04933	AGE-RAGE signaling pathway in diabetic complications	17/150	100/8076	2.66E-12	7.59E-11	3.48E-11
KEGG	hsa04625	C-type lectin receptor signaling pathway	17/150	104/8076	5.15E-12	1.32E-10	6.08E-11
KEGG	hsa05161	Hepatitis B	20/150	162/8076	1.33E-11	2.86E-10	1.31E-10
KEGG	hsa04917	Prolactin signaling pathway	14/150	70/8076	2.50E-11	4.94E-10	2.26E-10
KEGG	hsa04014	Ras signaling pathway	23/150	232/8076	3.42E-11	6.29E-10	2.88E-10
KEGG	hsa04931	Insulin resistance	16/150	108/8076	1.06E-10	1.60E-09	7.32E-10
KEGG	hsa05163	Human cytomegalovirus infection	22/150	225/8076	1.26E-10	1.70E-09	7.82E-10
KEGG	hsa04010	MAPK signaling pathway	25/150	294/8076	1.26E-10	1.70E-09	7.82E-10
KEGG	hsa05145	Toxoplasmosis	16/150	112/8076	1.85E-10	2.38E-09	1.09E-09
KEGG	hsa04151	PI3K-Akt signaling pathway	27/150	354/8076	2.50E-10	3.06E-09	1.41E-09
KEGG	hsa05167	Kaposi sarcoma-associated herpesvirus infection	20/150	193/8076	3.33E-10	3.90E-09	1.79E-09
KEGG	hsa04066	HIF-1 signaling pathway	15/150	109/8076	1.21E-09	1.20E-08	5.50E-09
KEGG	hsa04722	Neurotrophin signaling pathway	15/150	119/8076	4.22E-09	3.61E-08	1.66E-08
KEGG	hsa05162	Measles	16/150	139/8076	4.80E-09	3.85E-08	1.77E-08
KEGG	hsa05210	Colorectal cancer	13/150	86/8076	5.04E-09	3.93E-08	1.80E-08
KEGG	hsa05235	PD-L1 expression and PD-1 checkpoint pathway in cancer	13/150	89/8076	7.76E-09	5.87E-08	2.69E-08
KEGG	hsa05212	Pancreatic cancer	12/150	76/8076	1.21E-08	8.89E-08	4.08E-08
KEGG	hsa04210	Apoptosis	15/150	136/8076	2.69E-08	1.87E-07	8.56E-08
KEGG	hsa04015	Rap1 signaling pathway	18/150	210/8076	5.52E-08	3.38E-07	1.55E-07
KEGG	hsa05218	Melanoma	11/150	72/8076	7.18E-08	4.19E-07	1.92E-07
KEGG	hsa05223	Non-small cell lung cancer	11/150	72/8076	7.18E-08	4.19E-07	1.92E-07
KEGG	hsa04510	Focal adhesion	17/150	201/8076	1.61E-07	8.47E-07	3.88E-07
KEGG	hsa04071	Sphingolipid signaling pathway	13/150	119/8076	2.67E-07	1.32E-06	6.06E-07
KEGG	hsa04012	ErbB signaling pathway	10/150	85/8076	3.44E-06	1.42E-05	6.53E-06
KEGG	hsa05221	Acute myeloid leukemia	9/150	67/8076	3.53E-06	1.42E-05	6.53E-06
KEGG	hsa04664	Fc epsilon RI signaling pathway	9/150	68/8076	4.01E-06	1.56E-05	7.16E-06
KEGG	hsa05214	Glioma	9/150	75/8076	9.14E-06	3.41E-05	1.56E-05
KEGG	hsa04370	VEGF signaling pathway	8/150	59/8076	1.17E-05	4.23E-05	1.94E-05
KEGG	hsa04072	Phospholipase D signaling pathway	11/150	148/8076	9.06E-05	2.88E-04	1.32E-04
KEGG	hsa05211	Renal cell carcinoma	7/150	69/8076	2.73E-04	7.88E-04	3.61E-04
KEGG	hsa05230	Central carbon metabolism in cancer	7/150	70/8076	2.98E-04	8.50E-04	3.90E-04
KEGG	hsa05206	MicroRNAs in cancer	14/150	310/8076	0.002	0.004	0.002
KEGG	hsa04630	JAK-STAT signaling pathway	9/150	162/8076	0.003	0.007	0.003
KEGG	hsa04919	Thyroid hormone signaling pathway	7/150	121/8076	0.007	0.015	0.007

**Table 6:** GO enrichment analysis of potentially relevant targets of AS-IV anti-SA-AKI.

**Ontology**	**ID**	**Description**	**GeneRatio**	**BgRatio**	***p* value**	**FDR**	***q* value**
BP	GO:0071216	cellular response to biotic stimulus	30/165	23 6/18670	2.99E-26	1.27E-22	5.26E-23
BP	GO:0032496	response to lipopolysaccharide	33/165	330/18670	2.03E-25	3.21E-22	1.32E-22
BP	GO:0050727	regulation of inflammatory response	38/165	485/18670	2.26E-25	3.21E-22	1.32E-22
BP	GO:0002237	response to molecule of bacterial origin	33/165	343/18670	7.08E-25	7.55E-22	3.11E-22
BP	GO:0071222	cellular response to lipopolysaccharide	27/165	205/18670	4.25E-24	3.62E-21	1.49E-21
BP	GO:0002446	neutrophil mediated immunity	37/165	499/18670	7.35E-24	5.22E-21	2.16E-21
BP	GO:0071219	cellular response to molecule of bacterial origin	27/165	212/18670	1.06E-23	6.48E-21	2.67E-21
BP	GO:0072593	reactive oxygen species metabolic process	28/165	284/18670	1.85E-21	9.83E-19	4.06E-19
BP	GO:0042119	neutrophil activation	34/165	498/18670	8.71E-21	3.84E-18	1.59E-18
BP	GO:0050900	leukocyte migration	34/165	499/18670	9.28E-21	3.84E-18	1.59E-18
CC	GO:0031983	vesicle lumen	30/166	339/19717	3.61E-22	1.03E-19	7.75E-20
CC	GO:0060205	cytoplasmic vesicle lumen	29/166	338/19717	4.53E-21	6.44E-19	4.87E-19
CC	GO:0034774	secretory granule lumen	28/166	321/19717	1.48E-20	1.40E-18	1.06E-18
CC	GO:1904813	ficolin-1-rich granule lumen	16/166	124/19717	7.59E-15	5.39E-13	4.07E-13
CC	GO:0101002	ficolin-1-rich granule	18/166	185/19717	2.27E-14	1.29E-12	9.74E-13
CC	GO:0045121	membrane raft	20/166	315/19717	2.47E-12	1.06E-10	8.03E-11
CC	GO:0098857	membrane microdomain	20/166	316/19717	2.62E-12	1.06E-10	8.03E-11
CC	GO:0098589	membrane region	20/166	328/19717	5.18E-12	1.84E-10	1.39E-10
CC	GO:0005775	vacuolar lumen	14/166	172/19717	2.17E-10	6.83E-09	5.17E-09
CC	GO:0090575	RNA polymerase II transcription factor complex	11/166	163/19717	1.37E-07	3.90E-06	2.95E-06
MF	GO:0004879	nuclear receptor activity	14/166	47/17697	6.12E-18	1.53E-15	1.02E-15
MF	GO:0098531	transcription factor activity, direct ligand regulated sequence-specific DNA binding	14/166	47/17697	6.12E-18	1.53E-15	1.02E-15
MF	GO:0003707	steroid hormone receptor activity	13/166	56/17697	3.61E-15	6.02E-13	4.02E-13
MF	GO:0019902	phosphatase binding	18/166	185/17697	1.38E-13	1.73E-11	1.16E-11
MF	GO:0004175	endopeptidase activity	25/166	427/17697	2.45E-13	2.45E-11	1.64E-11
MF	GO:0008236	serine-type peptidase activity	17/166	182/17697	1.33E-12	1.11E-10	7.42E-11
MF	GO:0017171	serine hydrolase activity	17/166	186/17697	1.90E-12	1.36E-10	9.07E-11
MF	GO:0004252	serine-type endopeptidase activity	16/166	160/17697	2.19E-12	1.37E-10	9.16E-11
MF	GO:0019903	protein phosphatase binding	15/166	140/17697	4.01E-12	2.23E-10	1.49E-10
MF	GO:0004713	protein tyrosine kinase activity	14/166	134/17697	3.03E-11	1.52E-09	1.02E-09

**Table 7:** KEGG enrichment analysis of potentially relevant targets of AS-IV anti-SA-AKI.

**Ontology**	**ID**	**Description**	**GeneRatio**	**BgRatio**	***p* value**	**FDR**	***q* value**
KEGG	hsa05418	Fluid shear stress and atherosclerosis	24/150	139/8076	3.87E-17	9.95E-15	4.57E-15
KEGG	hsa05205	Proteoglycans in cancer	25/150	205/8076	3.74E-14	4.81E-12	2.21E-12
KEGG	hsa05215	Prostate cancer	18/150	97/8076	1.19E-13	9.19E-12	4.22E-12
KEGG	hsa01522	Endocrine resistance	18/150	98/8076	1.43E-13	9.19E-12	4.22E-12
KEGG	hsa05133	Pertussis	16/150	76/8076	3.73E-13	1.92E-11	8.79E-12
KEGG	hsa05135	Yersinia infection	20/150	137/8076	5.46E-13	2.34E-11	1.07E-11
KEGG	hsa01521	EGFR tyrosine kinase inhibitor resistance	16/150	79/8076	7.08E-13	2.60E-11	1.19E-11
KEGG	hsa04657	IL-17 signaling pathway	17/150	94/8076	9.25E-13	2.97E-11	1.36E-11
KEGG	hsa04933	AGE-RAGE signaling pathway in diabetic complications	17/150	100/8076	2.66E-12	7.59E-11	3.48E-11
KEGG	hsa04625	C-type lectin receptor signaling pathway	17/150	104/8076	5.15E-12	1.32E-10	6.08E-11
KEGG	hsa04659	Th17 cell differentiation	17/150	107/8076	8.30E-12	1.94E-10	8.90E-11
KEGG	hsa05161	Hepatitis B	20/150	162/8076	1.33E-11	2.86E-10	1.31E-10
KEGG	hsa04917	Prolactin signaling pathway	14/150	70/8076	2.50E-11	4.94E-10	2.26E-10
KEGG	hsa04014	Ras signaling pathway	23/150	232/8076	3.42E-11	6.29E-10	2.88E-10
KEGG	hsa05142	Chagas disease	16/150	102/8076	4.33E-11	7.41E-10	3.40E-10
KEGG	hsa04660	T cell receptor signaling pathway	16/150	104/8076	5.87E-11	9.42E-10	4.32E-10
KEGG	hsa04931	Insulin resistance	16/150	108/8076	1.06E-10	1.60E-09	7.32E-10
KEGG	hsa05163	Human cytomegalovirus infection	22/150	225/8076	1.26E-10	1.70E-09	7.82E-10
KEGG	hsa04010	MAPK signaling pathway	25/150	294/8076	1.26E-10	1.70E-09	7.82E-10
KEGG	hsa05145	Toxoplasmosis	16/150	112/8076	1.85E-10	2.38E-09	1.09E-09
KEGG	hsa04151	PI3K-Akt signaling pathway	27/150	354/8076	2.50E-10	3.06E-09	1.41E-09
KEGG	hsa05167	Kaposi sarcoma-associated herpesvirus infection	20/150	193/8076	3.33E-10	3.90E-09	1.79E-09
KEGG	hsa05130	Pathogenic Escherichia coli infection	20/150	197/8076	4.82E-10	5.39E-09	2.47E-09
KEGG	hsa05152	Tuberculosis	19/150	180/8076	6.98E-10	7.27E-09	3.34E-09
KEGG	hsa05131	Shigellosis	22/150	246/8076	7.07E-10	7.27E-09	3.34E-09
KEGG	hsa04066	HIF-1 signaling pathway	15/150	109/8076	1.21E-09	1.20E-08	5.50E-09
KEGG	hsa04668	TNF signaling pathway	15/150	112/8076	1.79E-09	1.70E-08	7.81E-09
KEGG	hsa04062	Chemokine signaling pathway	19/150	192/8076	2.10E-09	1.93E-08	8.84E-09
KEGG	hsa05219	Bladder cancer	10/150	41/8076	2.48E-09	2.19E-08	1.01E-08
KEGG	hsa04722	Neurotrophin signaling pathway	15/150	119/8076	4.22E-09	3.61E-08	1.66E-08
KEGG	hsa05120	Epithelial cell signaling in Helicobacter pylori infection	12/150	70/8076	4.57E-09	3.79E-08	1.74E-08
KEGG	hsa05162	Measles	16/150	139/8076	4.80E-09	3.85E-08	1.77E-08
KEGG	hsa05210	Colorectal cancer	13/150	86/8076	5.04E-09	3.93E-08	1.80E-08
KEGG	hsa05235	PD-L1 expression and PD-1 checkpoint pathway in cancer	13/150	89/8076	7.76E-09	5.87E-08	2.69E-08
KEGG	hsa05212	Pancreatic cancer	12/150	76/8076	1.21E-08	8.89E-08	4.08E-08
KEGG	hsa04926	Relaxin signaling pathway	15/150	129/8076	1.30E-08	9.28E-08	4.26E-08
KEGG	hsa04210	Apoptosis	15/150	136/8076	2.69E-08	1.87E-07	8.56E-08
KEGG	hsa05132	Salmonella infection	20/150	249/8076	2.86E-08	1.93E-07	8.86E-08
KEGG	hsa04217	Necroptosis	16/150	159/8076	3.37E-08	2.22E-07	1.02E-07

### Astragalus and AS-IV enhance the PI3K/AKT pathway in both *in vivo* and *in vitro*

**Figure 5:**
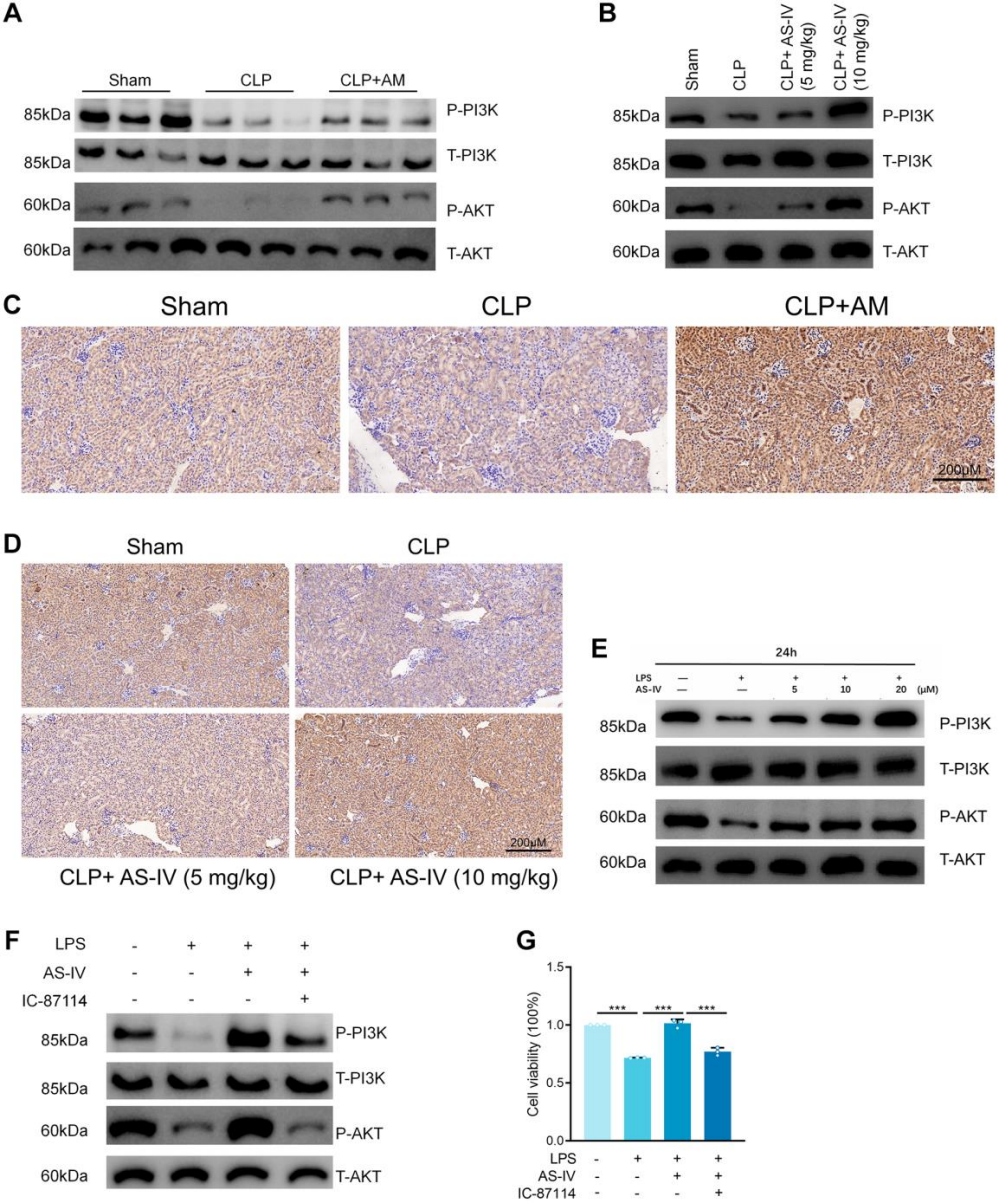
**Astragalus and AS-IV enhanced PI3K/AKT pathway *in vivo* and *vitro*.** (**A** and **B**) Expression level of Phospho-PI3K, PI3K, Phospho-AKT and AKT in kidney sections collected at 24 hours after CLP surgery following treatment with (250 mg/kg) Astragalus membranaceus or low (5 mg/kg) or high dosage (10 mg/kg) AS-IV. (**C** and **D**) Representative images showing Phospho-AKT immunohistochemical staining of mice kidney tissues. (**E**) Expression level of Phospho-PI3K, PI3K, Phospho-AKT and AKT in HK-2 cells incubated with LPS (1 μg/ml) plus AS-IV at the concentrations of 5, 10, 20 μM for 6 hours. (**F**) Protein expression of Phospho-PI3K, PI3K, Phospho-AKT and AKT in HK-2 cells incubated with LPS (1 μg/ml) plus AS-IV (20 μM) or PI3K inhibitor IC-87114 (5 μM) for 6 hours. (**G**) MTT results for AS-IV (20 μM) or PI3K inhibitor IC-87114 (5 μM) treatment on HK-2 cells incubated with LPS (1 μg/ml) for 6 hours. Data are presented as the mean ± SEM (*n* = 3 per group, ^*^P < 0.05, ^**^P < 0.01, ^***^P < 0.001).

Gene enrichment analysis revealed that the PI3K/AKT pathway was enriched for the most corresponding target genes, indicating that PI3K/AKT is an important intracellular pathway, and a previous study confirmed that PI3K/AKT plays an important role in SA-AKI [15, 16]. Western Blot analysis revealed that PI3K and AKT phosphorylation was inhibited in the apical kidney tissues of the mice model when compared to the sham-operated group and that PI3K and AKT phosphorylation was significantly increased in the astragalus and AS-IV administration group when compared to the CLP group in a dose-dependent manner (Figure 5A, 5B and Supplementary Figure 3A, 3B). This confirms the hypothesis in the preceding section on network pharmacology that Astragalus and AS-IV are involved in the regulation of the PI3K/AKT pathway. Additionally, immunohistochemistry was used to determine the phosphorylation levels of AKT in the kidney tissues. The results indicated that AKT phosphorylation was inhibited in the apoptotic kidney tissues of the mice model when compared to the sham-operated group, but was significantly elevated in the Astragalus and AS-IV group compared to the CLP group (Figure 5C and 5D). Furthermore, Western Blot analysis revealed that LPS suppressed PI3K and AKT phosphorylation relative to the normal group, but AS-IV significantly elevated PI3K and AKT phosphorylation compared to the LPS group in a dose-dependent manner (Figure 5E and Supplementary Figure 3C). To further establish the involvement of PI3K/AKT, we used the PI3K inhibitor IC-87114 (5 μM) in HK-2 cells followed by incubation with LPS and AS-IV (20 μM) and found that phosphorylation was inhibited (Figure 5F and Supplementary Figure 3D). Additionally, the MTT assay confirmed that inhibiting the PI3K/AKT pathway decreased the cell survival rate induced by AS-IV (Figure 5G), corroborating our network pharmacology prediction that AS-IV was involved in regulating the PI3K/AKT pathway.

## DISCUSSION

Chinese medicine is an important treasure of traditional Chinese medicine and is the material basis for the treatment of diseases in Chinese medicine. However, because of its complex composition and diverse effects, it cannot be precisely located in the body. Modern medical research has discovered that new drug development, is not only time-consuming and costly but also requires the application of network pharmacology and bioinformatics to significantly reduce the difficulties in new drug development. New drugs with significant efficacy against septic acute kidney injury and minimal adverse effects and cytotoxicity are urgently needed, and some monomers derived from Chinese herbal medicines are better choices than the herbs themselves because they can be administered in smaller doses, and have better-expected effects [12, 17].

Astragalus membranaceus is a widely used Chinese herb known for its ability to tonify Qi and invigorate blood. In this web-based pharmacology study, a total of 22 preferred compounds with good oral bioavailability and drug-like coefficients were identified using the ADME parameters OB (>30%) and DL (>0.18) reference values. Additionally, a review of the literature revealed that astragaloside analogues, the active components of Astragalus membranaceus, protect against sepsis-induced by cecum ligation perforation by inhibiting the inflammatory response and upregulation of the protein C pathway and that astragaloside has a protective effect on the kidney. Therefore, the present network pharmacology investigation included saponin components such as AS-I, AS-II, and AS-IV [18]. By establishing a compound-target network, 17 preferred Astragalus compounds against SA-AKI were identified based on network topology parameters. These compounds were then combined with a literature search, and we found that isorhamnetin [19], kaempferol [20], formononetin [21], Mairin [22], Jaranol [23], and Calycosin [24] had been reported in the literature in sepsis or acute kidney injury. Calycosins are flavonoids that have been shown to improve the pathological parameters associated with LPS-induced acute kidney injury, including lowering blood creatinine and urea nitrogen levels and reducing the expression of inflammatory factors, possibly by acting on the TLR4/NF-κB pathway to protect renal function and maintain normal kidney status [25]. Isorhamnetin, another flavonoid, has been investigated as a prospective option for bacterial infectious sepsis [19]. Kaempferol has been shown to protect septic mice against CLP-induced acute lung injury by inhibiting oxidative stress, iNOS, and ICAM-1 pathways [26]. Formononetin protects against cisplatin-induced AKI by activating the PPARα/Nrf2/HO 1/NQO1 pathway, promoting renal tubular cell proliferation, and inhibiting apoptosis following cisplatin-induced AKI [27]. Therefore, nine components were evaluated for their ability to induce septic acute kidney injury in HK-2 cells using LPS *in vitro*. Our findings indicated that that formononetin, AS-I and AS-IV, isorhamnetin, and Calycosin all exerted protective effects against LPS-induced cytotoxicity in HK-2 cells at 50 μM dosage, with AS-IV exerting the most prominent protective effect. Therefore, we investigated the mechanism of action of AS-IV against septic acute kidney injury both *in vitro* and *in vivo*.

AS-IV is the main active component of Astragalus and is frequently used in Chinese medicine as a quantitative and qualitative indicator of Astragalus. In recent years, various studies and reports have been published on the pharmacological effects of AS-IV *in vitro* and *in vivo*, including anti-oxidative stress, anti-inflammation, anti-apoptosis, and anti-fibrosis [18]. Numerous *in vivo* and *in vitro* studies have demonstrated that AS-IV has a high level of biological activity and can protect cells by inhibiting the production of epithelial-mesenchymal transition-related proteins, attenuating oxidative stress damage, inhibiting the release of inflammatory factors and other mechanisms [28]. AS-IV has been shown in previous studies to protect the nervous system and inhibit tumor disease progression by inhibiting endoplasmic reticulum stress [29] and to effectively reduce endoplasmic reticulum stress-induced renal tubular epithelial cell injury, podocyte autophagy, and endothelial cell inflammatory response, as well as to improve diabetic nephropathy injury [30]. In the present study, we found that LPS exposure significantly decreased HK-2 cell viability. AS-IV therapies, on the other hand, dose-dependently improved the viability of LPS-induced HK-2 cells in the model group. Similar to *in vitro* studies, in septic rats, AS-IV therapies markedly decreased CLP-induced tubular damage and improved tubular pathology. PI3K is an important inositol and phosphatidylinositol kinase. Three subtypes of PI3K have been identified: class I, class II, and class III, with class I being the most extensively investigated. PI3K-IA is a receptor-mediated dimeric protein with dual protein kinase and lipid-like kinase activity [31], while PI3K-IB is a receptor-mediated and delivered G protein-coupled receptor [32]. Protein kinase B (protein kinase B, PKB/AKT) is a serine/threonine kinase that regulates a variety of cellular biological activities, including cell cycle, proliferation, and apoptosis [33]. It also plays a key role in the PI3K/AKT pathway [34]. The PI3K/AKT signaling pathway is closely associated with the inflammatory response. Inhibiting this pathway significantly decreases the expression of inflammatory vesicles containing the Nod-like receptor protein 3 (NLRP3), which in turn significantly decreases the release of the downstream inflammatory factor IL-1β [35]. Lysosomes respond to their damaged organelles and macromolecules in response to hypoxia, infection, or starvation. The PI3K/AKT pathway is an important signaling pathway involved in this process [36]. Wullschleger et al. discovered a strong correlation between the PI3K/AKT pathway and inflammation [37]. Inhibition of the PI3K/AKT pathway resulted in a significant decrease in the release of the inflammatory factor NLRP3. Inhibition of the PI3K/AKT pathway decreased NLRP3 expression, which resulted in a significant decrease in IL-1β release. While the current study did not establish a direct and irreversible relationship between PI3K/AKT and sepsis-associated AKI, further exploration of its pathogenesis and prevention offers new possibilities for early diagnosis and treatment of the disease. Evidence suggests that AS-IV may have cardioprotective effects via altering the PI3K/AKT pathway and activating autophagic flow [38]. AS-IV has been demonstrated to improve cardiac function and decrease myocardial hypertrophy in several *in vivo* studies by decreasing oxidative stress and activating calpain-1, inhibiting the TBK1/PI3K/AKT signaling pathway, and several other additional mechanisms. Additionally, AS-IV can exert angiogenic and cardioprotective effects on the myocardium following myocardial infarction by activating the PTEN/PI3K/AKT signaling pathway and modulating the PI3K/AKT/GSK-3β pathway to alleviate the I/R effects caused by ligation of the left anterior descending coronary artery in rat hearts [39]. This study demonstrated that Astragalus and AS-IV treatment attenuated renal tubular epithelial cell injury in CLP mice and LPS-induced HK-2 cells, perhaps via a mechanism involving PI3K/AKT activation. However, this study only demonstrated the protective effect of LPS-induced tubular injury in HK-2 cells and CLP mice, although the function and mechanism of other pathogenic factors such as hyperglycemia and hyperlipidemia, on renal tubular epithelial cells need to be investigated further.

In summary, our findings demonstrate for the first time that Astragalus membranaceus and AS- IV protect against sepsis-induced AKI in renal tubular epithelial cells by enhancing the PI3K/AKT pathway. This study provides evidence that Astragalus membranaceus and AS- IV may be a significant therapeutic approach in the treatment of sepsis-induced AKI.

## Supplementary Materials

Supplementary Materials and Methods

Supplementary Figures 1-3
