# Supplementary material for: Protective effect of Astragalus membranaceus and Astragaloside IV in sepsis-induced acute kidney injury
Source: Aging (Albany NY). 2022 Jul 20;14(14):5855–77. doi: 10.18632/aging.204189 (PMC9365550; doi:10.18632/aging.204189)
Supplement: Supplementary Materials and Methods [file aging-14-204189-s001.pdf]

SUPPLEMENTARY MATERIALS AND METHODS

High performance liquid chromatography (HPLC) analysis

The chromatographic column was an Agilent C18 column (4.6 mm × 250 mm, 5 μm); the mobile phase was acetonitrile-water (40:60); the flow rate was 1.0 mL/min; the detector was an ELSD6000; the drift tube temperature was 100°C; the carrier gas pressure was 200 kPa. 16.23 mg of Astragaloside standard was weighed precisely in a 50 mL volumetric flask, and the solution was dissolved with methanol and diluted to the scale. The extract was recovered from the solvent and concentrated to dryness, added 10 mL of water to dissolve, and transferred to a separating funnel. The extract was extracted with 40 mL of water-saturated n-butanol four times with shaking, and the n-butanol solution was combined and washed twice with 40 mL of ammonia test solution. The residue was dissolved with methanol, transferred to a 10 mL volumetric flask, fixed with methanol and shaken well to obtain the test solution. The sample solution was extracted by 0.45 μm microporous membrane, and the average peak area was determined according to the above chromatographic conditions.

Serum creatinine and blood urea nitrogen assay

Serum creatinine and blood urea nitrogen was determined according to the manufacturer's instruction.

Enzyme-linked immunosorbent assay (ELISA)

Concentration of IL-6 and IL-1β in serum were measured by ELISA kit (Abcam) in consonance with standard protocols.

Cell viability assay

Cells were seeded into 96-well plates. After attachment, cells were incubated with or without AS-IV for another 15 min, and then incubated with or without LPS for another 6 h. After treatment, cells were incubated with 500 μg/ml 3-(4,5-dimethyl-thiazol-2-yl) 2,5-diphenyltetrazolium bromide (MTT) for 4 h. The formed formazan in surviving cells was dissolved in 10% SDS–5% iso-butanol–0.01 M HCl, and the optical density was measured at 570 nm with 630 nm as a reference. Cell viability was calculated as the percentage of control.
