# Supplementary material for: Protective effect of Astragalus membranaceus and Astragaloside IV in sepsis-induced acute kidney injury
Source: Aging (Albany NY). 2022 Jul 20;14(14):5855–77. doi: 10.18632/aging.204189 (PMC9365550; doi:10.18632/aging.204189)
Supplement: Supplementary Figures 1-3 [file aging-14-204189-s002.pdf]

SUPPLEMENTARY FIGURES

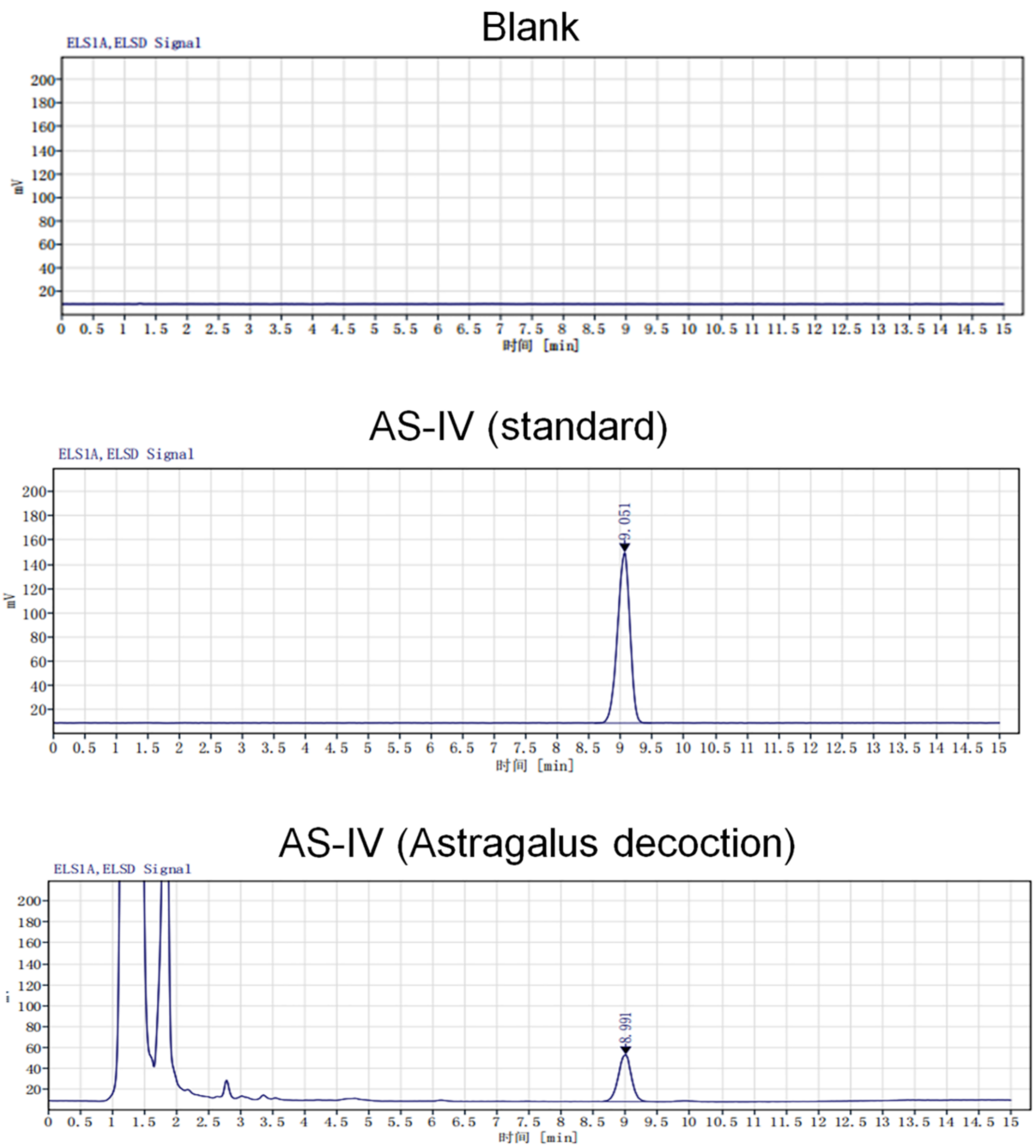

Supplementary Figure 1. HPLC profiles of AS-IV in blank, standard or Astragalus decoction.

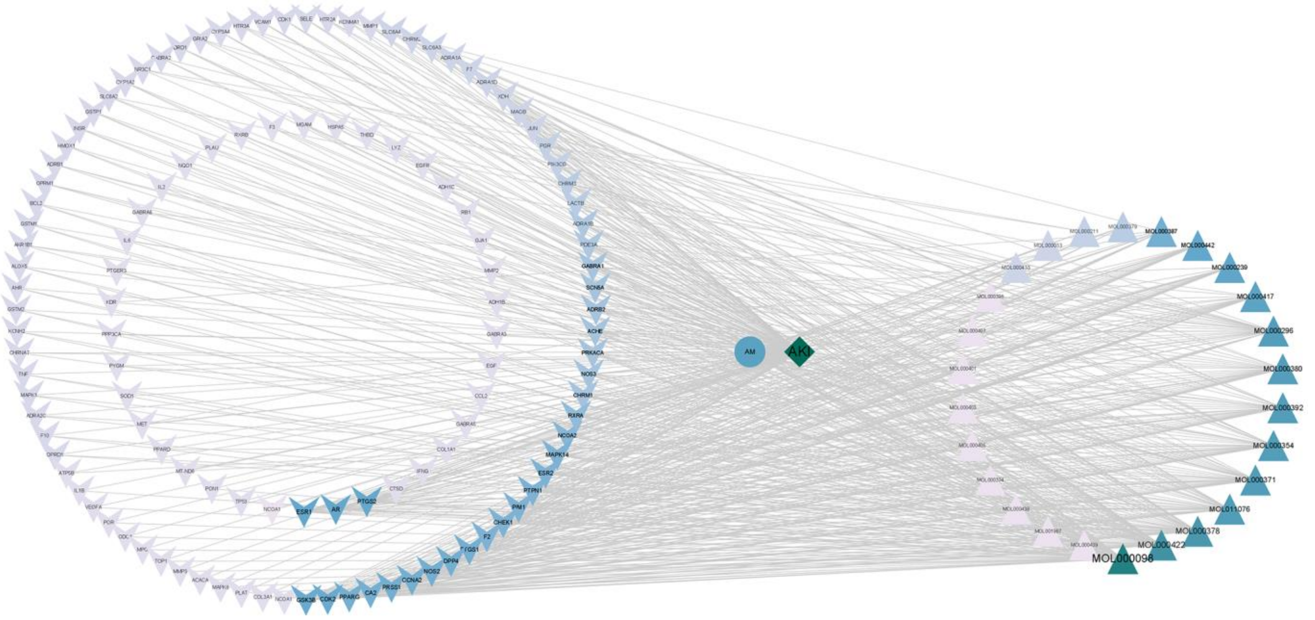

Supplementary Figure 2. Network of active compounds, core targets, and pathway of AM against SA-AKI.

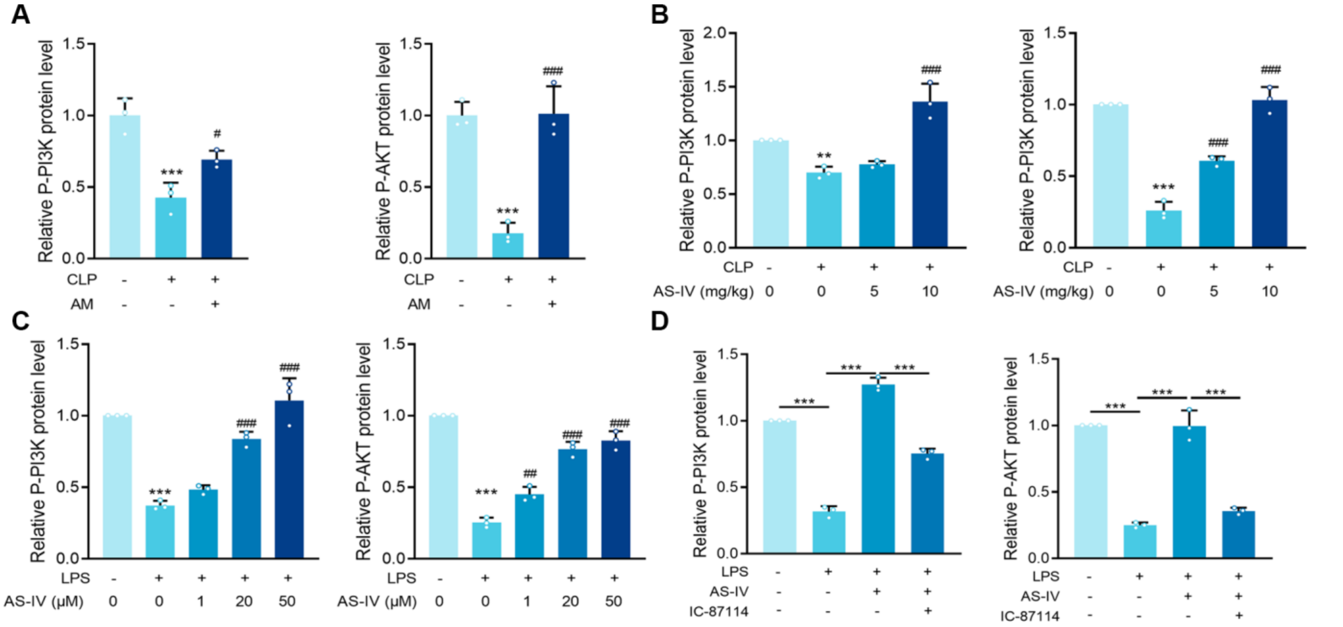

Supplementary Figure 3. (**A**) Relative P-PI3K/T-PI3K and P-AKT/T-AKT protein level in mice kidney tissues. (**B**) Relative P-PI3K/T-PI3K and P-AKT/T-AKT protein level in mice kidney tissues. (**C**) Relative P-PI3K/T-PI3K and P-AKT/T-AKT protein level in HK-2 cells. (**D**) Relative P-PI3K/T-PI3K and P-AKT/T-AKT protein level in HK-2 cells. Data are presented as the mean ± SEM (n = 6 per group, $^{**}P < 0.01$, $^{***}P < 0.001$ compared to Control group, $^{\#}P < 0.05$, $^{\#\#}P < 0.01$, $^{\#\#\#}P < 0.001$ compared to CLP or LPS group).
